# Autophagy and Exosomes Relationship in Cancer: Friends or Foes?

**DOI:** 10.3389/fcell.2020.614178

**Published:** 2021-01-12

**Authors:** Marta Colletti, Donatella Ceglie, Angela Di Giannatale, Francesca Nazio

**Affiliations:** Department of Pediatric Hemato-Oncology and Cell and Gene Therapy, IRCCS, Bambino Gesù Children’s Hospital, Rome, Italy

**Keywords:** autophagy, exosome, microenvironment, cancer, target therapy

## Abstract

Autophagy is an intracellular degradation process involved in the removal of proteins and damaged organelles by the formation of a double-membrane vesicle named autophagosome and degraded through fusion with lysosomes. An intricate relationship between autophagy and the endosomal and exosomal pathways can occur at different stages with important implications for normal physiology and human diseases. Recent researches have revealed that extracellular vesicles (EVs), such as exosomes, could have a cytoprotective role by inducing intracellular autophagy; on the other hand, autophagy plays a crucial role in the biogenesis and degradation of exosomes. Although the importance of these processes in cancer is well established, their interplay in tumor is only beginning to be documented. In some tumor contexts (1) autophagy and exosome-mediated release are coordinately activated, sharing the molecular machinery and regulatory mechanisms; (2) cancer cell-released exosomes impact on autophagy in recipient cells through mechanisms yet to be determined; (3) exosome-autophagy relationship could affect drug resistance and tumor microenvironment (TME). In this review, we survey emerging discoveries relevant to the exosomes and autophagy crosstalk in the context of cancer initiation, progression and recurrence. Consequently, we discuss clinical implications by targeting autophagy-exosomal pathway interaction and how this could lay a basis for the purpose of novel cancer therapeutics.

## Autophagy: An Overview

Autophagy is a self-degradative process occurring in all eukaryotic cells for maintaining homeostasis and cell survival. In basal conditions, autophagy degrades cytosolic materials such as long-lived proteins and old organelles for renewal of cellular components. During stressful conditions such as starvation or hypoxia, autophagy is induced to recycle macromolecules, providing energy and nutrients. Autophagy begins with the formation of a unique smooth double-membrane phagophore that traps cytosolic materials; after closure, autophagosome vesicle is formed and upon fusion with lysosomes, the inner membrane and the cargos are degraded and subsequently recycled (Yu et al., 2018). Three morphologically and mechanistically distinct types of autophagy have been described: macroautophagy (here referred to as autophagy), microautophagy and chaperone-mediated autophagy (CMA) (Abdrakhmanov et al., 2020). They differ substantially for cargo selection and delivery mechanism into lysosomes: macroautophagy is characterized by the formation of autophagosome, while during microautophagy the cargos are sequestered by direct invagination of the vacuole membrane. CMA only occurs in mammalian cells and uses chaperones to identify cargo proteins that contain a particular pentapeptide motif that are translocated directly across the lysosomal membrane. Although autophagy was initially thought to be a non-selective degradation mechanism, now it is clear that it allows the removal of specific cellular components such as mitochondria (mitophagy), aggregates (aggrephagy), or invading pathogens (xenophagy) (Gatica et al., 2018).

Autophagosome formation is driven by the autophagy-related (ATG) proteins that are both spatially and temporally controlled and are divided into distinct units: ULK complex, the class III phosphatidylinositol 3-kinase (PI3K) complex, the ATG2-ATG18/WIPI4 complex, ATG9, the ATG12 conjugation system and the ATG8/LC3 conjugation system (Nishimura and Tooze, 2020).

The role of autophagy has been explored in many fields (Yang and Klionsky, 2020). In cancer progression, autophagy has a dual and paradoxical role: while at early stages autophagy acts as a tumor suppressor mechanism, in advanced stages it has a fundamental role in tumor survival being active in response to cellular stress (White, 2015; Keulers et al., 2016; Cotzomi-Ortega et al., 2018; Amaravadi et al., 2019, 2020; Mulcahy Levy and Thorburn, 2020).

## Exosomes: Biogenesis, Release and Functions

According to the International Society for Extracellular Vesicles (ISEV) which provides guidelines for the classification of extracellular vesicles (EVs), exosomes are nano-sized (30–120 nm) (Théry et al., 2018). EVs secreted by all cell types that play a role in cell-cell communication through the transfer of active biomolecules such as proteins, lipids, RNAs, DNA and microRNAs (Raposo and Stoorvogel, 2013). Exosome precursors, named intraluminal vesicles (ILVs), derive from the membrane of endocytic cisternae by inward budding of microdomains. Upon ILVs accumulation, the cisternae become multivesicular bodies (MVBs) that undergo exocytic fusion with the plasma membrane followed by release of their ILVs to the extracellular space (Raposo and Stoorvogel, 2013; Cocucci and Meldolesi, 2015). Due to the mechanism of biogenesis, the exosomal membrane has the same orientation as the parental cell plasma membrane, and it is enriched in endosome-related proteins, lipids and tetraspanins. The exact mechanisms involved in exosomes packaging have not been fully elucidated but their secretion requires formation of an endosomal-sorting complex that is required for transport (ESCRT) (Scita and Di Fiore, 2010). ESCRT is comprised of four complexes (ESCRT−0, −I, −II, and −III) and associated proteins (vacuolar protein sorting-associated protein 4 (VPS4), tumor Susceptibility 101 (Tsg101) and ALIX) (Schuh and Audhya, 2014). In addition to ESCRT, which recognizes ubiquitylated proteins, other ESCRT-independent mechanisms operate to generate exosomes (Stuffers et al., 2009). These unconventional ESCRT-independent pathways seem to be driven by the presence of certain lipids, such as ceramides and lysobisphosphatidic acid (Matsuo et al., 2004; Babst, 2011). The release of exosomes into the extracellular environment requires the transport and docking of MVBs as well as their fusion with the plasma membrane (van Niel et al., 2006). These processes need several factors including molecular switches, cytoskeleton, molecular motors and the membrane fusion apparatus. It has been proposed that exosome release is a Ca^2+^-dependent (Savina et al., 2003) and pH-dependent (Parolini et al., 2009) process. After secretion, exosomes can be transferred to recipient cells *via* clathrin-mediated endocytosis (Tian et al., 2014), lipid raft-mediated endocytosis (Svensson et al., 2013), heparin sulfate proteoglycans-dependent endocytosis (Christianson et al., 2013), or phagocytosis (Feng et al., 2010). These pathways lead to different sorting and fate of exosomal cargo and the route by which exosomes are internalized appears to be cell and context specific. Tian et al. (2014) have showed that clathrin-mediated endocytosis and macropinocytosis are involved in the up-take of PC12-derived exosomes through a receptor-mediated mechanism. Svensson et al. (2013) have demonstrated that the signaling status of recipient cells is important in determining the pathway by which exosomes are internalized: exosomes derived from glioblastoma (GBM) cells, indeed, trigger lipid raft-mediated endocytosis where ERK activation is required. Furthermore, Christianson and co-workers provide evidences that exosomes produced by GBM cells require heparan sulfate proteoglycans for internalization and this affects the functional effects of exosomes in cancer cells (Christianson et al., 2013). Feng et al. (2010) have highlighted the role of the type of recipient cell in determining how exosomes are internalized: they have showed that phagocytic cells internalize exosomes *via* phagocytosis while in non-phagocytic cells exosomes attach to the cell membrane. In target cells, molecules carried by exosomes can trigger and influence several processes both in physiological and pathological conditions. In recent years, numerous evidence highlights the involvement of exosomes in angiogenesis promotion (Skog et al., 2008; Hong et al., 2009; Ahmadi and Rezaie, 2020), suppression of immune response (Yu et al., 2007; Clayton et al., 2008), induction of invasive (Luga et al., 2012; Guo et al., 2019; Jabbari et al., 2020a) and metastatic phenotype (Peinado et al., 2012), formation of pre-metastatic niche (Costa-Silva et al., 2015; Colletti et al., 2020). Moreover, tumoral exosomes can induce drug resistance carrying miRNAs that target antiapoptotic and immune-suppressive pathways or ABC transporters, which export chemotherapeutic agents out of recipient cells (Santos and Almeida, 2020). Given their involvement in cancer progression and their presence in different biological fluids, there have been increasing efforts toward their characterization as a source of possible diagnostic and prognostic biomarkers even in pediatric oncology (Colletti et al., 2017, 2019, 2020; Galardi et al., 2019, 2020) and as a delivery tool for biomedical applications (Rezaie et al., 2018; Rahbarghazi et al., 2019; Jabbari et al., 2020b; Wu Z. et al., 2020).

## Crosstalk Between Autophagy and Exosome Biogenesis

**FIGURE 1:**
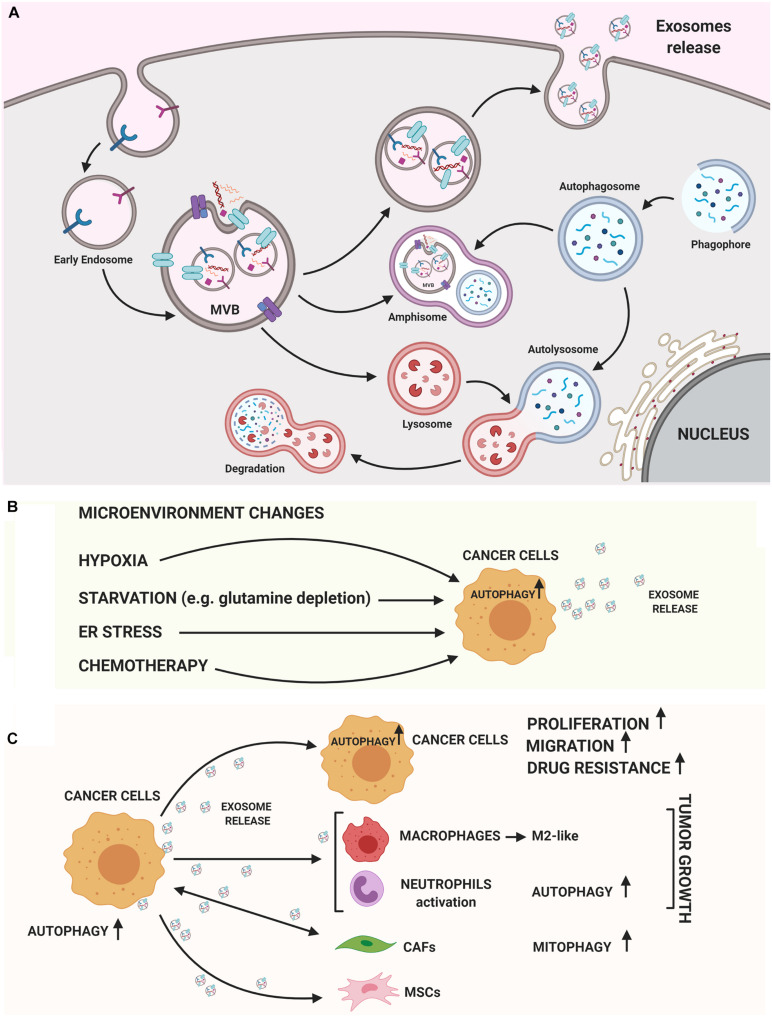
Autophagy and exosomes relationship. **(A)** A crosstalk between exosome biogenesis and autophagy flux occurs both at molecular level and at membranous vesicles such as amphisomes. In the cytoplasm several Rab-GTPase proteins regulate the movement of vesicles between autophagy and exosomal secretory pathway. On the MVB’s membrane different autophagic proteins such as LC3B, ATG5, and ATG16L1 participate to generate exosomes. Then, exosomes can carry autophagic cargo and secrete this into extracellular milieu. **(B)** Both autophagy and exosome release are strongly stimulated by TME conditions (hypoxia, starvation, ER stress) or chemotherapy treatments. **(C)** Exosomes released by cancer cells can induce autophagy in recipient cells, stimulating growth, migration and enhancing drug resistance. On the other hand, cancer cell-released exosomes can activate pro-tumoral stromal or immune cells *via* autophagy-related mechanisms or MSCs-derived exosomes may induce oncogenic autophagy in recipient cancer cells. The figure was performed with https://biorender.com.

An intricate relationship among autophagy and the exosome biogenesis (Figure 1A) occurs at different stages (Buratta et al., 2020; Hassanpour et al., 2020; Salimi et al., 2020). If in some cellular contexts autophagy and exosome production act at the same time to counter cellular stress (Kumar et al., 2014), in other circumstances the two processes can compensate each other. In fact, dysfunctional MVBs can be degraded by autophagy and the inhibition of lysosomal function or autophagy restores exosome secretion (Villarroya-Beltri et al., 2016). Moreover, EVs can have a cytoprotective role by inducing intracellular autophagy and, on the other hand, autophagy regulates the biogenesis and degradation of EVs (Xu et al., 2018). Finally, emerging evidence supports a role of both autophagy and exosomes in contributing to the export of cytokines or proteins by an unconventional secretory pathway (Ponpuak et al., 2015; Zhang et al., 2015; Kimura et al., 2017). The main advances about the crosstalk between these pathways are summarized below.

### Molecular Interaction Mechanism

Some studies are emerging about how individual ATG proteins can regulate exosome biogenesis and secretion (Salimi et al., 2020). Intriguingly, it has been observed that ATG5, which participates at the stage of autophagosome precursor synthesis (Nishimura and Tooze, 2020), allows the dissociation of vacuolar proton pumps (V_1_V_0_-ATPase) from the MVBs, thus inhibiting the acidification of MVB-lumen and contributing to the fusion of MVB with the plasma membrane (Guo et al., 2017) in a canonical autophagy-independent manner. The treatment with V-ATP inhibitors of Atg5 knockout cells demonstrated that luminal pH plays a role in controlling whether MVBs must undergo fusion with lysosomes for degradation or with plasma membrane for exosomes release (Mauthe et al., 2018). Guo et al. (2017) have demonstrated that the down-regulation of both ATG16L1, a core autophagy protein implicated at distinct phases of autophagosome biogenesis (Nishimura and Tooze, 2020), and ATG5 reduces exosome biogenesis in breast cancer cells; this, in turn, decreases tumor metastasis. Moreover, G alpha interacting protein (GAIP) and GAIP interacting protein C-terminus (GIPC), two proteins initially identified for G-protein coupled receptor subunit GI alpha (De Vries et al., 1998), can simultaneously stimulate exosome biogenesis and autophagy flux in pancreatic tumor cells (Bhattacharya et al., 2014).

Murrow et al. (2015) have demonstrated that the inhibition of ATG12–ATG3, a complex essential for a late step of autophagosome formation (Nishimura and Tooze, 2020), changes the form of MVBs, disrupts late endosome trafficking and reduces exosome biogenesis. This occurs through an interaction between ATG12-ATG3 and ALIX, a protein implicated in membrane fission which interacts with ESCRT members involved in exosomes release. ALIX inhibition also reduces basal autophagy flux, indicating a reciprocal regulation between autophagy and exosome biogenesis. Moreover, loss of ALIX or depletion of ATG12-ATG3 complex does not impact on starvation-induced autophagy, specifying the association of different complexes which control basal and stress-induced autophagy (Murrow et al., 2015).

Interestingly, a study perfomed by Bader and collaborators reports that the transmembrane protein ATG9 is implicated in the formation of ILVs in *Drosophila melanogaster*. In basal conditions, depletion of ATG9 leads to both autophagy inhibition and decrease of the ILVs content of amphisomes and autolysosome (Bader et al., 2015).

One of the key autophagy players is MAP1LC3B, Microtubule Associated Protein 1 Light Chain 3 Beta (or LC3B). LC3B is one of the main autophagy flux markers: in the initiation step, LC3B conjugation complex induces autophagosome biogenesis through ULK activation; during the maturation step, LC3B mediates closure, fusion and transport of the autophagosome (Mizushima et al., 2011). LC3B is incorporated into autophagosome membranes but it is also recruited to single-membrane phagosomes in a process called LC3-associated phagocytosis (LAP), which does not require the formation of autophagosomes (Florey et al., 2011; Martinez et al., 2011). LC3B has been found into exosomes (LC3-I rather than LC3-II) in an ATG7-independent manner, suggesting that the LAP-like lipidation mechanism could share a non-degradative role in exosome secretion. Furthermore, a very recent work has identified a new secretory mechanism where components of LC3 conjugation complex favor the association with RNA binding proteins (RBPs) and small non-coding RNAs into EVs, resulting in their secretion outside of cells (Leidal et al., 2020). This process has been defined as LDELS: LC3-Dependent EV Loading and Secretion. Using a proximity-dependent biotinylation proteomics strategy, the authors found that this mechanism does not require canonical autophagy but only components of the LC3 conjugation machinery, linking exosome secretion pathway, extracellular RNA release and autophagy in a very fascinating way. Finally, although not designated as ATGs, soluble N-ethylmaleimide-sensitive factor attachment protein receptor (SNARE) proteins are also an example of the interplay between autophagy and exosome secretion (Zhao and Zhang, 2019). SNARE complexes (such as STX17-SNAP29-VAMP7/VAMP8 or STX7-SNAP29-YKT6) mediate autophagosome-lysosome fusion; however, secretory autophagy requires different SNAREs than degradative autophagy (such as Sec22b), adding further to the complexity of this crosstalk (Zhao and Zhang, 2019).

Interestingly, growing evidence indicates that exosomes could contain ATG proteins. For example, Sequestosome 1 (SQSTM1), a classical receptor of autophagy, Neighbor Of BRCA1 Gene 1 Protein (NBR1), a selective autophagy receptor, WD Repeat Domain, Phosphoinositide Interacting 2 (WIPI2), a component of the autophagy machinery, or LC3 were found into exosomal fractions in PC-3 cells; intriguingly, both SQSTM1 and CD63, used as a representative exosomal marker protein, were found in the same MVB-like organelles inside the cells (Hessvik et al., 2016). Minakaki et al. (2018) have discovered that, in neuronal cells, autophagy inhibition increases alpha-synuclein levels in EVs released in human cerebrospinal fluid. These vesicles are biochemically characterized by the presence of both LC3-II and SQSTM1 together with classical MVB-EV markers. This study provides, for the first time, the presence of EVs with a hybrid autophagosome-exosome-like profile.

### Vesicular Interaction Mechanism

Antagonist interaction between autophagy and exosomes release has been well-defined in the context of amphisomes biogenesis. Amphisomes are degradative hybrid compartments formed after fusion between autophagosomes and MVBs, which can then fuse with lysosomes (Liou et al., 1997). As an example, autophagy induction supports MVB-autophagosome fusion and leads to a reduction of exosomes release (Fader et al., 2008). On the other hand, autophagy inhibition rescues exosome secretion, suggesting an involvement of autophagy in the lysosome-dependent degradation of MVBs (Villarroya-Beltri et al., 2016).

Recently, using biochemical, electron microscopy and fluorescence microscopy-based approaches, Ariotti et al. (2020) dissect a novel autophagy-based secretion of Caveolin 1 (CAV1) in addition to conventional exosome-based release. In details, in pancreatic cancer cells, they identified a novel class of exosomes, enriched in CAV1 (50–60 copies), and released *via* a non-canonical secretory autophagy pathway.

## Autophagy and Exosomes Relationship in Cancer

Although the importance of autophagy and exosomes in tumor progression is well-documented (Yu et al., 2007; Clayton et al., 2008; Skog et al., 2008; Hong et al., 2009; Svensson et al., 2013; Keulers et al., 2016; Ahmadi and Rezaie, 2020; Amaravadi et al., 2020), in recent years the understanding of their connection and interplay in cancer has aroused a lot of interest (Table 1; Kulshreshtha et al., 2007; Bellot et al., 2009; Chiavarina et al., 2010; Mazure and Pouysségur, 2010; White et al., 2010; Aga et al., 2014; Dutta et al., 2014; Li et al., 2016; Zhang et al., 2018, 2019; Jin et al., 2019; Meng et al., 2019; Shao et al., 2019; Wang et al., 2019, 2020; Yeon et al., 2019; Yuwen et al., 2019; Zeng et al., 2019; Zhou et al., 2019; Dai et al., 2020; Han et al., 2020; Huang et al., 2020; Kulkarni et al., 2020; Kumar and Deep, 2020; Liu et al., 2020; Sung et al., 2020; Wu X. et al., 2020; Yao et al., 2020).

**TABLE 1:** Summary of the exosomal molecules that regulate autophagy in target cells or whose release is regulated by autophagy in cancer models.

Exosomal molecule	Target molecule/pathway	Cell releasing exosomes	Target cells	Autophagy in cells releasing exosomes	Autophagy in target cells	Effect	References
miR-1910-3p	MTMR3	Breast cancer	Cancer cells	n.d.	Induction	Increased proliferation and migration	Wang et al., 2020
miR-1229-5p, miR-1246, miR-21-5p, miR-96-5p	Autophagy pathway	Serum of CRC patients	n.d.	n.d.	n.d.	Chemoresistance	Jin et al., 2019
miR-454-3p	ATG12	Serum of glioma patients; glioma cells	Glioma cells	n.d.	Suppression	Tumor suppression	Shao et al., 2019
miR-567	ATG5	Breast cancer	Cancer cells	n.d.	Suppression	Increased trastuzumab sensitivity	Han et al., 2020
miR-425-3p	AKT1	NSCLC	Cancer cells	n.d.	Induction	Increased platinum chemoresistance	Yuwen et al., 2019
miR-30a	BECLIN1/Bcl2	OSCC	Cancer cells	n.d	Induction	Decreased cisplatin sensitivity	Kulkarni et al., 2020
miR-19a-3p	phosphatase and tensin homolog/AKT/mTOR signaling pathway	SHSY5Y (NB cells)	Microglia cells	n.d.	Suppression	Dysfunction of autophagy in recipient cells	Zhou et al., 2019
CircNRIP1	AKT1/mTOR	Gastric cancer	Cancer cells	n.d.	Suppression	Increased tumor progression	Zhang et al., 2019
Circ-PVT1	miR-30a/YAP1	Gastric cancer	Cancer cells	n.d	Induction	Increased cisplatin chemoresistance	Yao et al., 2020
WNT1	WISP-3	CT26^*Flag–CAGE1*^ (mouse colon cancer cells)	CT26, mast cells and macrophages	Increased	Induction	Increased tumorigenic potential	Yeon et al., 2019
HMGB1	TLR4/NF-kB pathway	Gastric cancer	Neutrophils	n.d.	Induction	Increased pro-tumor activation of neutrophils	Zhang et al., 2018
n.d.	n.d.	Breast cancer	Mammary epithelial cells	n.d.	Induction	ROS production, DNA damage response, release of tumor promoting factors	Dutta et al., 2014
n.d.	ATG5	BMMSC	OS cells	n.d.	Induction	Promotion of proliferation, migration and invasion	Huang et al., 2020
LMP1	p65/NF-kB pathway	NPC cells	NFs, CAFs	n.d.	Induction	Promotion of proliferation, migration and radiation resistance of NPC cells	Wu X. et al., 2020
MALAT1	AKT1/mTOR	Lung carcinoma cells	DCs	n.d.	Induction	T cells proliferation inhibition	Liu et al., 2020
KRASG12D	STAT3 pathway	PDAC	Macrophages	Increased	n.d.	Polarization of macrophages into M2-like TAM	Dai et al., 2020
ITGB4	BNIP3L	Breast cancer	CAFs	n.d	Induction (Mitophagy)	Induced tumor progression	Sung et al., 2020
LC3, SQSTM1, SQSTM1–349, NBR1, NDP52	n.d.	Breast cancer cells	Breast cancer cells	Increased	Induction	Increased proliferation	Wang et al., 2019
VEGF,miR-9	n.d.	HUVEC	HCC cell lines	Decreased	Induction	Increased angiogenesis	Zeng et al., 2019
n.d.	n.d.	Adenocarcinoma cells	Adenocarcinoma cells	n.d.	Induction	Chemoresistance	Li et al., 2016

*MTMR3, myotubularin related protein 3; CRC, colorectal cancer; NSCLC, non-small cell lung cancer; OSCC, oral squamous cell carcinoma; NB, neuroblastoma; YAP-1, yes associated protein-1; WISP-3, WNT1-inducible-signaling pathway protein 3; HMGB1, high mobility group box 1; TLR4, toll like receptor 4; BMSC, bone marrow mesenchymal stem cells; OS, osteosarcoma; LMP1, latent membrane protein 1; NPC, nasopharyngeal carcinoma; NFs, normal fibroblasts; CAFs, cancer associated fibroblasts; MALAT1, metastasis associated lung adenocarcinoma transcript 1; DCs, dendrytic cells; PDAC, pancreatic ductal adenocarcinoma; ITGB4, integrin beta 4; BNIP3L, BCL2 interacting protein 3 like; SQSTM1, sequestrome1; VEGF, vascular endothelial growth factor; HUVEC, human umbilical vein endothelial cells; HCC, hepatocellular carcinoma; n.d., not determined.*

### Are Autophagy and Exosome-Mediated Release Coordinately Induced During Carcinogenesis?

In tumor cells, both autophagy and exosome release are strongly activated, suggesting that both these pathways are a part of cancer cells response (Figure 1B). This coordinated activation may represent an adaptive stress response, although the molecular details are not yet understood. Hypoxic tumor microenvironment (TME) is a key feature in many solid tumors and it is associated with unfavorable prognosis. In tumors, both starvation and hypoxia induce autophagy, which avoids inflammation and cell death (Bellot et al., 2009; Chiavarina et al., 2010; Mazure and Pouysségur, 2010; White et al., 2010). Many studies have shown that cancer cells secrete a higher number of exosomes under hypoxic conditions (Kumar and Deep, 2020) and hypoxia is able to alter the proteomic and nuclear acid profiles of exosomes (Meng et al., 2019). Interestingly, HIF-1α was found in exosomes with transcriptional activity (Aga et al., 2014), representing a potential cancer biomarker. In addition, several miRNAs under the transcriptional control of HIF-1α are enriched in EV derived from hypoxic cells; among these, miR-23a targets BCL2 Interacting Protein 3 Like (BNIP3L), a crucial mitophagy receptor (Kulshreshtha et al., 2007). Moreover, high levels of the HIF1-α transcriptional target *BCL2 Interacting Protein 3 (BNIP3)* mRNA, another mitophagy receptor, are found in EV produced by hypoxic glioma cells (Kucharzewska et al., 2013).

In cancer cells autophagy and exosome release may be concomitantly up-regulated in response to other cellular stressors such as unfolding protein response (UPR) and endoplasmic reticulum (ER) stress. ER stress is known to increase autophagy in several types of normal and tumor cells (Verfaillie et al., 2010; Corazzari et al., 2017). Kanemoto et al. (2016) found that MVB formation and exosomes release are enhanced by ER stress; furthermore, the down-regulation of both Inositol-Requiring Protein 1 (IRE1α) and PKR-like ER kinase (PERK), two key players of UPR pathway, impacts on exosome production. In addition, the spliced form of X-box binding protein 1 (sXBP1), a key transcription factor that promotes UPR, was found in exosomes, suggesting the transmission outside the cell of UPR mechanism, following exposure to stresses (Hosoi et al., 2018).

Very recently, using *Drosophila* model and human cell lines, Fan et al. (2020) have found that glutamine depletion induces secretion of exosomes carrying exclusive cargos created in Rab11−positive recycling endosomal MVBs. Interestingly, the release of exosomes from glutamine depleted HCT116 cells stimulate angiogenesis and enhances tumor cell proliferation. Glutamine depletion is likely to be an autophagy inducer determining tumor growth (Tan et al., 2017), supporting the concept of a strict connection between autophagy and exosome secretion as a part of neoplastic cells response.

Beside microenvironmental conditions, up-regulation of both autophagy and exosome release has been well-recognized after chemotherapy treatments (Bandari et al., 2018; Yun and Lee, 2018; Ab Razak et al., 2019; Kang et al., 2020) and in mediating chemoresistance (Yun and Lee, 2018; Ender et al., 2019; Nazio et al., 2019; Steinbichler et al., 2019). For example, exosomes can carry functional plasma membrane transporter proteins from resistant cells to their drug-sensitive ones (Lu et al., 2013) or can sequestrate drugs reducing their concentration (Goler-Baron et al., 2012). It is unknown whether up-regulation of both autophagy and exosome secretion is part of the resistance mechanism or a consequence of cellular phenotype changes. Nevertheless, autophagy inhibiiton and modulation of exosome release may serve for therapeutic approaches and needs to be investigated.

### How Do Exosomes Released by Tumor Influence Autophagy in Recipient Cells?

Specific cancer exosomal miRNAs and proteins seem to have a crucial role in determining an ATG response (Figure 1C; Jin et al., 2019; Shao et al., 2019; Yuwen et al., 2019; Han et al., 2020; Kulkarni et al., 2020; Wang et al., 2020). Recently, exosomes carrying miR-1910-3p secreted by breast cancer cells have been found to promote tumor development inducing proliferation, migration and autophagy in recipient mammary epithelial cells and breast cancer cells (Table 1; Wang et al., 2020). Several recent studies have shown that specific exosomal miRNAs regulate autophagy-dependent therapy resistance in recipient cells (Kaminskyy et al., 2012; Zhou et al., 2012; Dutta et al., 2014; Zhang et al., 2018; Deng et al., 2019; Huang et al., 2020; Kang et al., 2020). In breast cancer, exosomal miR-567 down-regulates ATG5 and consequently autophagy, reversing trastuzumab resistance (Dutta et al., 2014). In cisplatin-resistant non-small cell lung cancer (NSCLC), exosomal miR-425-3p down-regulates AKT1 inducing autophagy and leading to therapeutic failure both in early and advanced stages (Yuwen et al., 2019). Also, in oral squamous cell carcinoma (OSCC), exosomal miR-30a modulates cisplatin-sensitivity reducing autophagy *via* Beclin1 and Bcl2 (Kulkarni et al., 2020). These studies are in support for the use of exosome- mediated miRNA delivery as an effective therapeutic approach. In a very recent paper, exosomal circRNA-plasmacytoma variant translocation 1 (circ-PVT1) intensifies cisplatin-resistant gastric cells through modulating autophagy, invasion, and apoptosis; circ-PVT1 negatively controls miR-30a-5p that, in turn, regulates Yes-associated protein 1 (YAP1) levels (Yao et al., 2020). Besides general autophagy, there is some evidence that also specific forms of autophagy could be modulated by exosomes. Liu et al. (2019) showed that hepatitis B virus (HBV)-infected liver cancer cells-derived exosomes promote liver cancer chemoresistance by modulating the CMA pathway. Specifically, an increased expression of lysosome associated-membrane protein type 2A (LAMP2A), a membrane protein that acts as a specific receptor for the CMA, was observed in cells treated with HBV-associated exosomes and this is associated with a down-regulation of cell death after oxaliplatin treatment due to the activation of the CMA pathway. This is the first study investigating the connection between CMA and exosome release in cancer drug resistance; it proposes the targeting of exosomes to increase chemosensitivity in patients with HBV-liver cancer.

In addition to having a role on tumor cells communication, cancer cell-released exosomes are also able to modulate ATG mechanisms in surrounding stromal and immune cells to support tumor progression. In a study performed by Zhang et al. (2018) suggest that gastric cancer cell-derived exosomes induce autophagy and pro-tumor activation of neutrophils, which, in turn, promote gastric cancer cell migration. Other authors have begun to investigate the importance of exosomes-autophagy interplay between normal and neoplastic cells in supporting carcinogenesis. For example, mesenchymal stem cells (MSCs)-derived exosomes have been identified to modulate autophagy in pathological conditions such as during ischemia or spinal cord injury (Baixauli et al., 2014; Tian et al., 2019) and, more recently, in cancer (Huang et al., 2020). Huang et al. (2020) indeed, found that MSCs-derived exosomes promote osteosarcoma development and invasion by inducing autophagy.

Dai et al. (2020) found that extracellular KRAS^*G12D*^ is packaged into exosomes and transferred, through them, from cancer cells to macrophages. In pancreatic ductal adenocarcinoma (PDAC), G12D is the most frequent mutation in KRAS. In this work the authors demonstrate that oxidative-stress induced autophagy regulates KRAS^*G*12D^ protein release from PDAC cells, and this drives macrophages polarization into pro-tumor M2-like tumor-associated macrophages. Given that autophagy can influence exosome release, a novel study discovers a potential strategy to counteract esophangeal squamous cell carcinoma (ESCC) growth by affecting autophagy and exosome-mediated paracrine senescence (Zheng et al., 2020). Sulforaphane, an isothiocyanate derived from cruciferous vegetables, inhibited fusion process between autophagosome and lysosome resulting in significantly higher exosome release; these exosomes evidently trigger senescence of receipt ESCC cells in a ROS-mTOR-dependent manner. This is in line with the idea that defects in autophagy avoid the effective degradation of intracellular aggregates and exosome discharge may be increased to improve the proteotoxic stress. Another study proposes a link between mitochondria-selective autophagy and exosome content in cancer. Sung et al. (2020) reported that triple negative breast cancer-derived exosomal Integrin beta 4 (ITGB4) induces a metabolic reprogramming in cancer-associated fibroblasts (CAFs) that, in turn, supports tumor progression. Exosomal ITGB4 triggers the conversion of pyruvate to lactate in CAFs *via* BCL2 Interacting Protein 3 Like (BNIP3L)-dependent mitophagy. The produced lactate is released in the extracellular space and then taken-up by breast cells. This study suggests that ITGB4-induced mitophagy could be a novel target for cancer therapy.

### Unveiling Exosomal Contents as New Frontier for Autophagy Modulation and Cancer Treatment

In the era of precision medicine, the development of targeted drugs is also addressing several efforts in investigating new pharmaceutical compound that can modulate autophagy, overcoming the stress tolerance of the tumor and undermining the mechanism of survival of tumor cells. In cancer biology, autophagy plays dual role in both tumor promotion and suppression. In this context the choice to induce or inhibit autophagy is related to the role of autophagy in each specific cancer. A large number of clinical trials using autophagy inhibitors (Malhotra et al., 2019) or activators (Geissler et al., 2016; Rodríguez-Perálvarez et al., 2018; Kulka et al., 2020) are ongoing and, when used in association with anti-cancer drugs, can sensitize chemoresistant cells to treatment (Singh et al., 2018). Chloroquine/Hydroxychloroquine is the only autophagy inhibitor that has been approved by the FDA; however, it also has many off-target effects and the majority of clinical trials have been performed in patients with no specific selected criteria beyond the tumor type.

Given the close relationship between autophagy and exosome pathways in cancer, a better understanding of the biological basis of this complex dialog will help to design specific therapeutic strategy (Lin et al., 2019), such as nano-carriers therapy, to modulate autophagy. Although the use of nanotechnology for the delivery of drugs/biological products targeting autophagy is largely unexplored, investigation of exosome cargo contents could offer opportunities for affecting autophagy in a specific tumor context. In particular, for the treatment of personalized cancer, antagonistic oligonucleotides (antagomiRs, anti-miRs) may be designed for the development of autophagy-modulating therapy, increasing cell chemo-sensitivity and overcoming drug resistance. Proof-of-concept studies are required to understand the role of autophagy in each tumoral context and whether triggering or suppressing autophagy (by specific miRNAs/anti-MiRs) could counteract tumor aggressiveness and progression. To this regard, nanoparticles as miRNAs/miRs delivery systems for modulation of autophagy could be a promising therapeutic strategy.

## Conclusion

Autophagy and exosome pathways are strictly interconnected at several levels. In cancer, increasing evidence discussed above indicate a crucial interplay between these processes. Although exosomes control of autophagy is context-dependent, targeting the exosomal pathway to modulate autophagy may suggest a basis for aiming novel cancer therapeutics that need to be further studied. Moreover, the biomarker application of the regulatory factors of both autophagy and exosome signaling has been proposed. However, the effects of their interaction are intricate and TME-dependent and therefore need further valuations.

## Author Contributions

FN conceptualized the work and together with AD, DC, and MC prepared a draft of the manuscript text. MC contributed to the figures development. FN, AD, and MC critically reviewed and edited the manuscript. All authors discussed the reported information and commented on the manuscript.

## Conflict of Interest

The authors declare that the research was conducted in the absence of any commercial or financial relationships that could be construed as a potential conflict of interest.
